# Modeling and Simulation of the Role of Mass Testing in Controlling COVID-19

**DOI:** 10.1007/s11538-026-01593-8

**Published:** 2026-02-21

**Authors:** Alexandre Maranhão, Marco A. Ridenti, André J. Chaves

**Affiliations:** Department of Physics, Aeronautics Institute of Technology, Praça Marechal Eduardo Gomes, 50, 12228-900 São José dos Campos, SP Brazil

**Keywords:** Mathematical Epidemiology, Disease Models, COVID-19, Nonlinear Dynamics

## Abstract

This study explores the role of mass testing in controlling the COVID-19 pandemic using an age-stratified compartmental model. The model evaluates the impact of different testing strategies on the pandemic’s reproduction number, $R_0$, while also considering social distancing measures and demographic characteristics. The analysis highlights the importance of combining mass testing with isolation strategies to reduce the spread of the virus. The simulations demonstrate that in countries characterized by high levels of elderly cohabitation with younger individuals, vertical isolation is insufficient; horizontal isolation with work restrictions, alongside testing and susceptibility reduction measures, is crucial. For aged developed countries, where cohabitation of the elderly with younger individuals is less prevalent, and for least developed countries, where the population has a predominantly youthful age structure, pandemic control is more feasible with fewer tests. The study also emphasizes the critical role of identifying asymptomatic cases to achieve optimal epidemic control. Lastly, the cost-effectiveness of various testing strategies is examined, providing insights for public health policy decision-making.

## Introduction

Epidemiological modeling is a crucial tool for guiding public policies, with its analyses informing and assisting decision-making (Thompson 2020). During the 2019 Coronavirus (COVID-19) pandemic, governments worldwide have relied on mathematical models to understand the disease’s behavior across various scenarios and to predict the potential outcomes of different public policies [3, 2, 1]. For a model to be insightful, it must incorporate demographic and social interaction properties, especially when the goal is to assist decision-makers who must account for the unique characteristics of the population in question. Simultaneously, it is essential to connect the model’s output to practical strategies that can be implemented as viable public policies during health emergencies. Testing policies represent a specific type of public policy often considered among the strategies to mitigate or stop the spread of the disease. However, determining the optimal testing strategy and how best to implement it remains a challenging and non-trivial task.

After the COVID-19 pandemic, it became evident that catastrophic pandemics are more probable than previously assumed (Casadevall et al. 2024). In today’s interconnected world, the emergence of a mutant virus in any region can quickly lead to global spread due to air travel and other efficient means of human transportation (Muller and Nathan 2020). Simultaneously, viral genes will continue to mutate, potentially increasing the risks. Alarmingly, it is not far-fetched to imagine such scenarios coinciding with other catastrophic events, such as nuclear wars or natural disasters exacerbated by global warming (Muller and Nathan 2020; Casadevall et al. 2024; Caminade et al. 2019; Garry and Checchi 2020). In this context, the systematic study of initial actions to mitigate pandemics remains highly relevant (Okesanya et al. 2023; Lison et al. 2023). In the face of a new, unknown disease for which no vaccines or effective treatments exist, non-pharmaceutical interventions might be the only viable option to prevent catastrophic loss of human life (Okesanya et al. 2023; Lison et al. 2023). Providing accurate and actionable information to decision-makers is crucial for an effective pandemic response during such dire circumstances (Okesanya et al. 2023; Rubin et al. 2021). However, translating epidemiological modeling results into clear, well-founded public health strategies is often challenging (Lison et al. 2023; Rubin et al. 2021). This work aims to bridge the gap between mathematical modeling and public health policymaking, offering a method to better inform strategic responses to pandemics based on mass testing.

Mass testing has emerged as a central strategy in mitigating the spread of COVID-19, especially in the absence of effective pharmaceutical interventions during the early stages of the pandemic. Several countries implemented large-scale testing programs with varying levels of success, and numerous mathematical models have been developed to evaluate the effectiveness of such strategies.

Empirical studies have also underscored the potential and limitations of mass testing. For instance, Larremore (2021) demonstrated that the frequency of testing and rapid turnaround time may be more critical than test sensitivity in reducing transmission. The Slovak mass testing campaign provided a real-world example of population-scale screening. Pavelka (2021) reported that nationwide testing with antigen tests contributed to a temporary reduction in prevalence, though its long-term effectiveness was debated. In contrast, the UK’s Liverpool pilot program showed more modest outcomes (Raffle and Gill 2021).

From a policy perspective, Paltiel (2020) used modeling to assess testing strategies in university settings, arguing that frequent, low-cost testing could enable safe reopening. Similarly, Kretzschmar (2020) examined the impact of testing and contact tracing, finding that effectiveness depends strongly on delays and coverage.

Despite these advances, many existing models do not account for demographic heterogeneity, such as age structure, which plays a fundamental role in COVID-19 transmission dynamics and outcomes. Our study contributes to this gap by integrating mass testing into an age-stratified SEAHIRQ model, enabling a more nuanced evaluation of targeted testing policies across populations with distinct age and contact structures. Moreover, by incorporating varying intervention scenarios and demographic profiles, we aim to provide insights that are more directly applicable to diverse public health contexts.

This study extends a previously implemented age-stratified compartmental model (Ridenti et al. 2020, 2023) to simulate and analyze the effectiveness of mass testing policies in combating the COVID-19 pandemic. By incorporating demographic characteristics and social distancing measures, the model evaluates the impact of various testing strategies through the calculation of the reproduction number $R_0$, establishing a clear link between policy decisions and pandemic suppression. Consistent with earlier findings (Ridenti et al. 2023) and related studies (Davies et al. 2020a; Prem et al. 2017), the results underscore the significant influence of demographic and social interaction patterns on epidemic dynamics. To address these factors comprehensively, this study assessed the effects of different testing strategies across four distinct population profiles. Each profile represents unique demographic and economic characteristics, categorized as underdeveloped, developing, developed, or aging developed countries (Ridenti et al. 2023).

Having evaluated the impact of different testing strategies across diverse population profiles, the study further explores how model parameters representing testing rates can inform practical public policies. Specifically, it assesses the cost of achieving efficient testing rates and examines the opportunity cost of delaying the implementation of a testing policy. To provide a more comprehensive analysis, the simulations incorporate varying social isolation scenarios, highlighting how this practice can mitigate the costs of effective mass testing. By interlinking social isolation, testing strategies, and demographic characteristics, the study provides a holistic perspective on pandemic management tailored to the unique contexts of the countries analyzed.

Several simulations using different compartment models have been developed to analyze strategies for controlling the SARS epidemic (Gumel et al. 2004) and COVID-19 (Aronna et al. 2021; Nadim and Chattopadhyay 2020; Zlatić et al. 2020; Gharouni et al. 2022; Xu et al. 2023). While the SARS model relies on distinct parameter values reflective of that outbreak, new analyses tailored specifically to COVID-19 remain essential due to the unique characteristics of the virus. For example, the model presented in Aronna et al. (2021) provides a rigorous and comprehensive mathematical framework for analyzing testing strategies. However, it does not account for age stratification, a critical factor for understanding demographic-specific dynamics. Similarly, the COVID-19 model in Nadim and Chattopadhyay (2020) features robust mathematical analysis, including an analytical demonstration of a backward bifurcation, yet also lacks age stratification. In contrast, Zlatić et al. (2020) proposes a simpler epidemic model, concentrating on the production and availability of testing resources and exploring system dynamics based on these parameters. Despite their strengths, none of these studies incorporate the effects of demographics or social isolation measures, which are pivotal in understanding the interplay between testing strategies and public health outcomes.

Building on the previous studies that analyzed the control of epidemics through testing and isolation, two recent works have focused specifically on the efficacy of these strategies for COVID-19 (Gharouni et al. 2022; Xu et al. 2023). The first, by Gharouni et al., extends the traditional SIR model (Kermack 1927) by subdividing each compartment into tested and untested groups, assigning relative testing weights to each group. This allows for a detailed examination of different testing strategies, particularly in terms of stratified random designs versus targeted approaches (Gharouni et al. 2022). The second study, by Xu and colleagues, builds on Gharouni’s framework but adds complexity by accounting for changes in individuals’ disease states while awaiting test results (Xu et al. 2023). Both studies provide valuable insights, though they do not incorporate age stratification or social isolation measures—important factors that we address in our model.

The model proposed here differs fundamentally from these previous approaches, with its own strengths and limitations. One of the key distinctions is that our model defines the rate at which symptomatic and asymptomatic infected individuals are placed in quarantine. This rate is directly linked to the probability of testing positive for each group, denoted as *xA* for asymptomatic cases and *xI* for symptomatic cases. By applying Bayes’ Theorem, we establish a connection between these probabilities and the overall test probability, relating the model more closely to public health policies and the dynamics of real-world testing strategies.

While this model does not capture the subtle dynamics of individuals awaiting test results—an issue explored by Xu et al. (2023)—it remains valid as long as the testing strategy ensures a sufficiently high proportion of infected individuals test positive. Although the model does not explicitly account for false negatives, this can be indirectly assessed by considering the reduced likelihood of an infected individual testing positive. Another limitation is that quarantine only begins after test results are received, overlooking the possibility that some individuals may self-quarantine while awaiting results. This assumption could potentially lead to an underestimation of the impact of testing. However, the model’s inclusion of an asymptomatic compartment allows for a more comprehensive analysis of strategies targeting asymptomatic individuals, such as through contact tracing, which is often critical to the success of a testing policy.

A significant strength of this model is its incorporation of social isolation and demographic factors, achieved through social contact matrices and age-dependent parameters (Davies et al. 2020a; Ridenti et al. 2020). These elements address gaps in previous models, offering a more realistic representation of how different population segments respond to testing strategies. As demonstrated in the following analyses, the two parameters *xI* and *xA* – the probabilities of symptomatic and asymptomatic individuals testing positive – serve as crucial control variables. We calculate $R_0$ across various populations by varying these parameters in combination with different demographic profiles and intervention scenarios. These measures provide insight into how susceptible a population must be for effective epidemic control, offering a clearer picture of the dynamics of testing policies and their effectiveness across diverse demographic groups.

Moreover, beyond analyzing the basic reproduction number, the simulation explores how variations in *xI* and *xA* impact the number of deaths over the course of a 400-day epidemic. This analysis emphasizes the opportunity cost of delaying testing implementation and complements the $R_0$ calculations by highlighting the long-term consequences of policy timing.

Finally, the study examines the relationship between testing strategies for symptomatic and asymptomatic individuals, comparing different methods for sustaining testing efforts over time. By analyzing the number of tests required for each strategy, this part of the study aims to offer guidance on public policy decisions related to the scale and duration of testing initiatives.

## Model Description

### Compartmental Models and Basic Reproduction Number $R_0$

     The use of compartmentalized epidemiological models assumes that the population can be divided into homogeneous subgroups within which individuals are indistinguishable, so that the model parameters may vary from one group to another but not within the same group (van den Driessche 2002). The most significant criterion for modeling is to divide the population based on the stage of the disease the individuals are in, thus creating groups of susceptible, infected, and recovered individuals, for example. In the COVID-19 case, compartmentalization by age is necessary due to the significant variation in parameters such as hospitalization rate, susceptibility, and fatality across age groups (Davies et al. 2020a). Therefore, each compartment must represent a stage of the disease for a specific age group.

Driessche van den Driessche (2002) describes a generalized compartmental epidemic model as follows: let $x=(x_1, x_2,... x_m)$ represent the compartmentalization of the population, where each $x_i$ is the number of individuals in a given compartment. The model is defined by a set of non-negative initial conditions for each state, along with a system of equations in the form of the equation:

1 $$\begin{aligned} \dot{x_i} = f_i(x) = \mathscr {F}_i - \nu _i, \end{aligned}$$

where $\mathscr {F}_i$ represents the rate of new infections entering compartment *i* and $\nu _i$ represents other transitions between compartments. Typically, letters are used for each $x_i$ to clarify the meaning of the variables, such as *I* for a compartment of infected individuals and *S* for susceptible individuals.

The basic reproduction number, $R_0$, is defined as the number of secondary infections caused by the introduction of an infected individual into a population of susceptible individuals (Diekmann et al. 1990, 2010). If $R_0 < 1$, the disease-free equilibrium (DFE) is stable, and the introduction of an infected individual does not lead to an epidemic. However, if $R_0 > 1$, the DFE is unstable, and the spread of the disease is always possible (van den Driessche 2002). Control strategies for infectious diseases often aim at reducing $R_0$, which is a necessary condition for stopping an epidemic.

The determination of the $R_0$ value depends on the modeling approach, the assumed variables, and the parameter estimation mechanisms, which leads to discrepancies between different analyses for the same epidemic (Viceconte and Petrosillo 2020). The World Health Organization (WHO) estimates that its value is between 1.4 and 2.5 [41] for the global coronavirus pandemic. Meanwhile, a systematic review of 29 studies estimates an average of 2.87, with the lowest value of 0.76 in South Korea and the highest of 6.32 in France (Billah et al. 2020).

From a compartmental model, it is possible to obtain the reproduction number analytically using the next-generation matrix method, proposed by Diekmann et al. (1990, 2010); van den Driessche (2002). Given the model described by equations in the form of equation 1, where the compartments of active infections are ordered as the first *m* components, the matrices *F* and *V* of order *m* are defined such that $F_{ij} = \frac{\partial \mathscr {F}i}{\partial x_j}$ and $V_{ij} = \frac{\partial \nu _i}{\partial x_j}$. The matrix $FV^{-1}$ is called the next-generation matrix and has the property that entry (*i*, *j*) represents the rate at which infected individuals in compartment *j* will cause new infections in compartment *i*. The value of $R_0$ is the spectral radius of this matrix, which is defined as the maximum of the absolute values of the matrix’s eigenvalues.

Although $R_0<1$ is always a necessary condition for controlling an epidemic, it is not always sufficient, depending on the type of bifurcation encountered at $R_0=1$. Bifurcation is a change in the behavior of equilibrium with the smooth variation of a parameter, in this case, $R_0$. An endemic equilibrium point is defined as any stable solution that has non-zero infected compartments, meaning a population configuration in which the disease stabilizes without ceasing. Some models exhibit a behavior called forward bifurcation, that is, for $R_0<1$ there is no endemic equilibrium, and the DFE is the only possible equilibrium, while for $R_0>1$ there will be a stable endemic equilibrium. Thus, ensuring that $R_0<1$ is both a necessary and sufficient condition for the suppression of the disease in a model with forward bifurcation.

Other models exhibit a behavior called backward bifurcation: stable endemic equilibrium and DFE coexist for $R_0<1$ (Gumel 2012). Backward bifurcation has serious consequences for disease control. First, because even when the goal of $R_0<1$ is achieved, the disease can persist if the number of infected individuals is high enough, requiring further efforts to reduce $R_0$ to a critical value $R^*<R_0<1$ to effectively suppress the disease. Furthermore, as $R_0$ increases and crosses the threshold $R_0=1$, the number of infected individuals may experience a potentially catastrophic jump, unlike the gradual increase observed in forward bifurcation (Martcheva 2019). Finally, when forward bifurcation occurs, the introduction of infected individuals into a population with $R_0<1$ does not cause an outbreak, as the system will return to DFE. However, with backward bifurcation, even with $R_0<1$, the introduction of infected individuals can lead to a new outbreak (Brauer et al. 2019).

### Compartment Model

A modification of the model proposed by Davies et al. (2020a) will be used, with the addition of symptomatic, hospitalized, and quarantined individuals. The model has been used previously (Ridenti et al. 2020, 2023), but not with the use of testing and the quarantined compartment. The population is divided into eight health states: susceptible (S), exposed (E), asymptomatic (A), symptomatic (I), recovered (R), quarantined symptomatic ($Q_i$), quarantined asymptomatic ($Q_a$), and hospitalized (H). Additionally, an auxiliary group *C* is defined as the number of deaths from COVID, although it is not part of the population’s compartmentalization. Each state is divided into 16 age groups, represented by the subscript *i*, forming a total of 128 compartments. The differential equations of the SEAHIRQ model are presented in Equations 2 a 10.

2 $$\begin{aligned} \frac{d}{dt}S_{i}= &  \Lambda _{i} N_{i} - \mu _{eq,i} S_{i} - \sum _{j} \beta _{ij} \frac{S_{i}}{N_{i}} I_{j} \nonumber \\ &  - \sum _{j} \alpha _{j} \beta _{ij} \frac{S_{i}}{N_i} A_{j} - \sum _{j} \xi _{j} \beta _{ij} \frac{S_{i}}{N_i} E_{j}, \end{aligned}$$

3 $$\begin{aligned} \frac{d}{dt}E_{i}= &  \sum _{j} \beta _{ij} \frac{S_{i}}{N_i} I_{j} + \sum _{j} \alpha _{j} \beta _{ij} \frac{S_{i}}{N_i} A_{j} + \sum _{j} \xi _{j} \beta _{ij} \frac{S_{i}}{N_i} E_{j} \nonumber \\  &  - \mu _{eq,i}E_{i} - a_{i}E_{i}, \end{aligned}$$

4 $$\begin{aligned} \frac{d}{dt}A_{i}= &  (1-\rho _{i}) a_{i} E_{i} - \mu _{eq,i}A_{i} - \gamma ^{R,A}_{i} A_{i} - \gamma ^{Qa,A}_{i}A_{i}, \end{aligned}$$

5 $$\begin{aligned} \frac{d}{dt}H_{i}= &  \gamma ^{H}_{i} I_{i} + \gamma ^{H,Qi}_{i} Qi_{i} -\gamma ^{H,R}_{i} H_{i} - (\mu _{eq,i} + \mu _{cov,i}) H_{i}, \end{aligned}$$

6 $$\begin{aligned} \frac{d}{dt}I_{i}= &  \rho _{i} a_{i} E_{i} - \mu _{eq,i}I_{i} - \gamma ^{H}_{i} I_{i} - \gamma ^{R,I}_{i} I_{i} - \gamma ^{Qi,I}_{i}I_{i}, \end{aligned}$$

7 $$\begin{aligned} \frac{d}{dt}R_{i}= &  \gamma ^{R,I}_{i} I_{i} + \gamma ^{R,A}_{i} A_{i} + \gamma ^{R,Qi}_{i} Qi_{i} + \gamma ^{R,Qa}_{i} Qa_{i} + \gamma ^{H,R}_{i} H_{i} - \mu _{eq,i} R_{i}, \quad \qquad \end{aligned}$$

8 $$\begin{aligned} \frac{d}{dt}Qi_{i}= &  \gamma ^{Qi,I}_{i} I_{i} - \gamma ^{R,Qi}_{i} Qi_{i} - \gamma ^{H,Qi}_{i} Qi_{i} - \mu _{eq,i} Qi_{i}, \end{aligned}$$

9 $$\begin{aligned} \frac{d}{dt}Qa_{i}= &  \gamma ^{Qa,A}_{i} A_{i} - \gamma ^{R,Qa}_{i} Qa_{i} - \mu _{eq,i} Qa_{i}, \end{aligned}$$

10 $$\begin{aligned} \frac{d}{dt}C_{i}= &  \mu _{cov,i} H_i. \end{aligned}$$

**Fig. 1:**
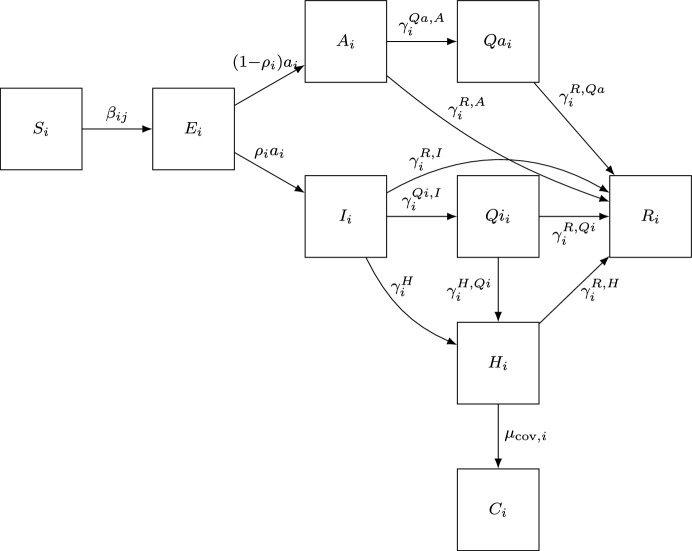
Schematic representation of the compartmental model. For simplicity, the birth rate and the death rate independent of COVID-19 are not represented in the diagram

In Eqs. 2 and 10 the parameters $\Lambda _i$ and $\mu _{eq,i}$ represent the birth and death rates of the population, independent of COVID-19. The parameters $\alpha _i$ and $\xi _i$ represent, respectively, the attenuation of infectivity by asymptomatic and pre-symptomatic individuals. The parameter $a_i$ is a conversion coefficient from exposed to infected, and $\rho _i$ is the probability that an exposed individual becomes symptomatic. The rates $\gamma$ refer to hospitalization of infected individuals ($\gamma ^{H}_i$) and quarantined infected individuals ($\gamma ^{H,Qi}_i$), recovery of hospitalized individuals ($\gamma ^{H, R}_i$), recovery of symptomatic and asymptomatic individuals, whether quarantined or not ($\gamma ^{R,I}_i$, $\gamma ^{R,A}_i$, $\gamma ^{R,Q_i}_i$, $\gamma ^{R,Q_a}_i$), and quarantine of symptomatic and asymptomatic individuals ($\gamma ^{Qi,I}_i$, $\gamma ^{Qa,A}_i$). The parameter $\mu _{cov,i}$ is the disease mortality rate (See Fig. 1).

The matrix $\beta _{ij}$ represents a contamination coefficient and is given by the product of the susceptibility vector $p_i$, that is, the probability that a contact results in an infection, and the contact matrix $C_{ij}$, which contains the number of daily contacts between people in age groups *i* and *j*. This matrix, in turn, is obtained by a transformation of the sum of the contact matrices at home ($C_{ij}^{H}$), at school ($C_{ij}^{S}$), at work ($C_{ij}^{W}$), and in other contexts ($C_{ij}^{O}$). The transformation *T* represents social isolation measures — for example, setting school contacts to zero,

11 $$\begin{aligned} \beta _{kj} = p_k \cdot T\left( C_{kj}^{H} + C_{kj}^{W} + C_{kj}^{S} + C_{kj}^{O} \right) = p_k \cdot \left( C_{kj}^{H} + C_{kj}^{W} + C_{kj}^{O} \right) \,\, . \end{aligned}$$

Thus, equation 11 describes how contacts are reflected in the matrix $\beta _{ij}$.

The effect of demography in the model is reflected in three ways: in the initial distribution of the population by age group, that is, the initial values of $S_0$ to $S_k$; in the different contact patterns of each country, which will be represented by different matrices $C_{i, j}$; and in different birth rates $\Lambda _i$ and death rates $\mu _{eq, i}$. This study will focus on the first two points, considering $\Lambda _i = \mu _{eq, i} = 0$.

### Properties of the Model

#### Local Stability of the Disease Free Equilibrium (DFE)

A solution is called disease free equilibrium (DFE) if the infected states are zero. For the specified model, all equations are reduced to the equations below:

12 $$\begin{aligned} 0= &  \Lambda _{i} N_{i} - \mu _{eq,i} S_{i} \,, \end{aligned}$$

13 $$\begin{aligned} 0= &  - \mu _{eq,i} R_{i} \,\,. \end{aligned}$$

In general, if $\mu _{eq,i}>0$, then $R_i=0$ and $S_i = \frac{\Lambda _i}{\mu _{eq, i}}N_i$. Thus, the DFE is

14 $$\begin{aligned} (S_i, E_i, A_i, H_i, I_i, R_i, Qa_i, Qi_i) = \left( \frac{\Lambda _i}{\mu _{eq, i}}N_i, 0, 0, 0, 0, 0, 0, 0 \right) \end{aligned}$$

for each *i*.

Since we considered $\Lambda _{i}=\mu _{eq,i}=0$, then $N_{i}$, $S_{i}$, and $R_{i}$ are free variables, that are not necessarily zero. Thus, the DFE is $(S_i, E_i, A_i, H_i, I_i, R_i, Qa_i, Qi_i) = (\alpha , 0, 0, 0, 0, \beta , 0, 0)$ for each *i*, with $\alpha$ and $\beta$ being non-zero positive values.

According to Theorem 2 of van den Driessche (2002), the DFE is locally asymptotically stable if $R_0<1$, and unstable if $R_0>1$.

#### Existence of Endemic Equilibrium

The variation of the population is given by

15 $$\begin{aligned} \frac{d}{dt}N_i = \Lambda _{i} N_{i} - \mu _{eq,i} N_{i} - \mu _{cov,i} H_i \,\, , \end{aligned}$$

which must be zero at equilibrium. If we consider $\Lambda _{i}=\mu _{eq,i}=0$, we arrive at

16 $$\begin{aligned} \mu _{cov,i} H_i = 0 \,\, . \end{aligned}$$

Since $\mu _{cov,i}>0$, it follows that $H_i=0$. For an equilibrium state, Eqs. 2 to 9 must equal zero. Substituting $H_i=0$ into Eq. 5, we find that $I_i=0$. Substituting $I_i=0$ into Eq. 6, we obtain $E_i=0$, and, from Eq. 8, we have $Qi_i=0$. Again, due to the parameters being positive, $E_i=0$ in Eq. 4 leads to $A_i=0$, which in turn leads Eq. 9 to $Qa_i=0$. Finally, the previous substitutions in Eq. 2 lead to $S_i=0$. Therefore, when ignoring birth rates, there is only the trivial solution, meaning the only stable equilibrium is the DFE.

One way to understand this phenomenon is that if there are no births, there must be no deaths to achieve equilibrium. Thus, no one can be hospitalized, and therefore, no one can be infected, exposed, and so on until reaching the DFE. The addition of births allows for endemic equilibria that this study will not analyze.

Therefore, for the case considered, the model does not exhibit forward or backward bifurcation, as there is no endemic equilibrium for either $R_0<1$ or $R_0>1$. The issue with having $R_0>1$ is that the DFE becomes unstable, and the introduction of infected individuals leads from one DFE to another, a process during which the infections and deaths that are to be avoided occur. Thus, control techniques can focus on achieving $R_0<1$.

## Simulation Methodology

### Intervention Scenarios

Among the intervention scenarios proposed by Ridenti et al. (2023), the scenarios of no intervention, vertical isolation, social distancing, and distancing with work restrictions were selected. The selection aims to provide epidemiological insights for the ongoing debate between vertical and horizontal distancing (Duczmal et al. 2020; Schuchmann et al. 2020), also comparing both with the raw scenario of no intervention. Furthermore, even when horizontal distancing is implemented, the inclusion of work restrictions is treated separately as it is related to the economy, and thus is among the arguments against horizontal isolation (Schuchmann et al. 2020).

#### 1: No intervention

This scenario has theoretical interest as it shows the trend of the pandemic if nothing is done to contain it. The contact matrix is given by

17 $$\begin{aligned} C_{kj} = C_{kj}^{H} + C_{kj}^{W} + C_{kj}^{S} + C_{kj}^{O} \,\, . \end{aligned}$$

#### 2: Elderly Isolation

Once the characteristics of the coronavirus have been identified, the solution of isolating the elderly arises naturally due to the higher susceptibility and lethality of the disease in this age group.

In order to restrict the contact of the elderly to within the household, a transformation is applied to reduce only the rows and columns representing the affected age groups. Some models also restrict contact within the household; however, while this may be possible in more developed countries, it is not the reality in countries like Brazil, where the elderly population often lives with and depends on younger individuals (Ridenti et al. 2023). The contact matrix is then given by

18 $$\begin{aligned} C_{kj} = C_{kj}^{H} + (\tilde{\textbf{I}} \cdot \textbf{C}^{W} \cdot \tilde{\textbf{I}} )_{kj} + ( \tilde{\textbf{I}} \cdot \textbf{C}^{S} \cdot \tilde{\textbf{I}})_{kj} +( \tilde{\textbf{I}} \cdot \textbf{C}^{O} \cdot \tilde{\textbf{I}})_{kj} \,\, , \end{aligned}$$

where $\tilde{\textbf{I}}$ is a diagonal matrix such that $I_{kk}=1$ if $k<i$ and $I_{kk}=P_k$ if $k\ge i$. Here, $i=12$ corresponds to the age group above 60 years, which is the first age group to be isolated, and $P_k$ represents the contact reduction for age group $k\ge i$. In the ideal case of perfect elderly isolation, $P_k$ would be zero, but that is not realistic. In this model, it is assumed that $P_k=0.1$, meaning that isolation reduces contact by 90% for any age group above 60 years.

#### 3: Social Distancing

Here, the combination of elderly isolation, school closures, and distancing in general, with a reduction in contacts, is considered.

In addition to restricting the contact of the elderly to within the household, this scenario zeros the contact matrix at school, reduces contacts in other contexts, and prescribes an increase in household contacts – greater increases for the young (0 to 20 years) and some increases for other groups. The final matrix is given by

19 $$\begin{aligned} C_{kj} = A_{kk} C_{kj}^{H} + (\tilde{\textbf{I}} \cdot \textbf{C}^{W} \cdot \tilde{\textbf{I}} )_{kj} + 0 \cdot ( \tilde{\textbf{I}} \cdot \textbf{C}^{S} \cdot \tilde{\textbf{I}})_{kj} + B_{kk} ( \tilde{\textbf{I}} \cdot \textbf{C}^{O} \cdot \tilde{\textbf{I}})_{kj} \,\, , \end{aligned}$$

where $\textbf{A}$ is a diagonal matrix, *e.g.*, $A_{ii} = 1.5$ for $i = 1$ to 4 and $A_{ii} = 1.1$ for $i > 4$, in the case of a 50% increase for the young and a 10% increase for other age groups; and $\textbf{B}$ is a diagonal matrix, e.g., $B_{ii} = 0.4$ for $i = 1$ to 4, and $B_{ii} = 0.6$ for $i > 4$.

We note that this transformation breaks the symmetry of the original contact matrix, since contact reductions are applied unequally across age groups. This asymmetry is intentional and reflects realistic scenarios where specific demographics (e.g., children during school closures, or the elderly under distancing recommendations) experience different behavioral or policy-driven changes in contact patterns. While contact matrices from empirical surveys are often symmetrized by modelers (Prem et al. 2017), raw data frequently exhibit asymmetries. In this work, we chose not to apply symmetrization—neither to the original matrices nor to those modified by intervention parameter. Importantly, this does not affect the validity of the next-generation matrix approach used to compute the basic reproduction number, as the theoretical framework allows for non-symmetric transmission matrices (Diekmann et al. 1990, 2010).

#### 4: Social Distancing with Work Restrictions

This scenario differs from the previous one because, in addition to imposing reductions in social contact in general, it also enforces rules regarding work. In addition to the effects described in the previous scenarios, there is a 40% reduction in workplace contacts ($\zeta _t=0.6$). The final matrix is given by

20 $$\begin{aligned} C_{kj} = C_{kj}^{H} + \zeta _{t} (\tilde{\textbf{I}} \cdot \textbf{C}^{W} \cdot \tilde{\textbf{I}} )_{kj} + 0 \cdot ( \tilde{\textbf{I}} \cdot \textbf{C}^{S} \cdot \tilde{\textbf{I}})_{kj} + B_{kk} ( \tilde{\textbf{I}} \cdot \textbf{C}^{O} \cdot \tilde{\textbf{I}})_{kj} \,\, . \end{aligned}$$

### Definition of the Model Parameters

We followed the method used in Ridenti et al. (2020) and Ridenti et al. (2023), obtaining hospitalization data from Verity et al. (2020) based on Chinese case statistics. The age-specific probabilities of a case manifesting clinically ($\rho _{i}$) and of a contact leading to infection (susceptibility $p_i$) were extracted from the work of Davies et al. (2020b).

The recovery rates $\gamma ^{R,I}_i$, $\gamma ^{R,A}_i$, $\gamma ^{H,R}_i$, and the conversion rate $a_i$ were obtained from the average infection durations ($\gamma ^{R,A}_i$ = $\gamma ^{R,I}_i \sim 1 - e^{-(1/d_{I})}$, with $d_{I}=5$ days (Woelfel et al. 2020)), hospitalization durations ($\gamma ^{H,R}_i \sim 1 - e^{-(1/d_{A})}$, $d_A=8$ days (Zhou et al. 2020)), and incubation periods ($a_i \sim 1 - e^{-(1/d_{L})}$, $d_L=7$ days (Lauer et al. 2020)).

The contact matrix values were obtained from a study that estimated the matrix for several countries (Prem et al. 2017). To analyze the relationship between demography and testing, we followed the method established by Ridenti et al. (2023): fictitious countries of 10 million inhabitants each were created using the contact matrices of Nigeria, Brazil, the United States, and Germany, representing, respectively, underdeveloped, developing, developed, and aging developed countries. These countries will hereafter be referred to as LDCs (Least Developed Countries), DGCs (Developing Countries), DCs (Developed Countries), and ADCs (Aged Developed Countries). For more information on the demographic characteristics of these country categories, we recommend consulting our previous work (Ridenti et al. 2023).

### Testing Parametrization

The testing parameters $\gamma ^{Q,I}_{i}$ and $\gamma ^{Q,A}_{i}$ are obtained from the ratios *xI* and *xA*, which are defined as the percentage of symptomatic and asymptomatic individuals to be tested, respectively, assuming that the test has 100% efficiency. The model also assumes that once the test result is positive, the patient will be isolated.

At the end of the epidemic, the ratio between the total number of entries into the quarantined infected compartment and the number of exits from the infected compartment should be *xI*, that is,

21 $$\begin{aligned} xI = \frac{ \int _{0}^{\infty } \gamma ^{Q,I}_{i} I(t),dt}{\int {0}^{\infty } (\gamma ^{Q,I}_{i}+\gamma ^{R,I}_{i}+\gamma ^{H}_{i})I(t)dt} = \frac{ \gamma ^{Q,I}_{i}}{\gamma ^{Q,I}_{i}+\gamma ^{R,I}_{i}+\gamma ^{H}_{i}} \,\, , \end{aligned}$$

22 $$\begin{aligned} xA = \frac{ \int _{0}^{\infty }\gamma ^{Q,A}_{i} A(t),dt}{\int {0}^{\infty } (\gamma ^{Q,A}_{i}+\gamma ^{R,A}_{i})A(t)dt} = \frac{ \gamma ^{Q,A}_{i}}{\gamma ^{Q,A}_{i}+\gamma ^{R,A}_{i}} . \end{aligned}$$

Thus, the effect of *xI* and *xA* on the parameters is given by

23 $$\begin{aligned} \gamma ^{Q,I}_{i} = \frac{xI \cdot (\gamma ^{H}_{i}+\gamma ^{R,I}_{i})}{1-xI} \,\, , \end{aligned}$$

24 $$\begin{aligned} \gamma ^{Q,A}_{i} = \frac{xA \cdot \gamma ^{R,A}_{i}}{1-xA}\,\, . \end{aligned}$$

The advantage of having such parameters is to map the rates $\gamma ^{Q,I}_i$ and $\gamma ^{Q,A}_i$, which may be difficult to determine, bijectively to rates that range between 0 and 1, simplifying the search space. Interpreting *xI* as the conditional probability of an infected person being tested $P(\textrm{Test}:\vert :I)$, and considering $P(\textrm{Test})$ as the probability of any individual being tested and $P(I:\vert :\textrm{Test})$ as the probability of a tested individual being symptomatic, Bayes’ Theorem can be applied to relate the definition of *xI* with public policy according to the equation

25 $$\begin{aligned} xI = P(\textrm{Test}:\vert :I) = \frac{P(\textrm{Test})\cdot P(I :\vert :\textrm{Test})}{P(I)} = \frac{N}{I} P(\textrm{Test})\cdot P(I :\vert :\textrm{Test}) \,\, , \end{aligned}$$

in which *P*(*I*) has been replaced by $\frac{I}{N}$. Doing the analogous for *xA*, we arrive at

26 $$\begin{aligned} xA = P(\textrm{Test}:\vert :A) = \frac{P(\textrm{Test})\cdot P(A :\vert :\textrm{Test})}{P(A)} = \frac{A}{I} P(\textrm{Test})\cdot P(A :\vert :\textrm{Test}). \end{aligned}$$

Thus, given a target *xI*, the number of tests required can be determined by

27 $$\begin{aligned} P(\textrm{Test}) = \frac{I}{N} \cdot \frac{xI}{P(I :\vert :\textrm{Test})} = \frac{A}{N} \cdot \frac{xA}{P(A :\vert :\textrm{Test})} \,\, , \end{aligned}$$

where the term $\frac{I}{N}$ is given by the model, and the remaining terms can be set as targets.

If individuals are tested randomly, $P(I :\vert :\textrm{Test}) = P(I)$, $P(A :\vert :\textrm{Test}) = P(A)$, and $xI = xA = P(\textrm{Test})$. However, testing can prioritize individuals with suspected symptoms – in this case, $P(I :\vert :\textrm{Test})$ is expected to be higher than the distribution of symptomatic individuals across the population as a whole. By setting a target $P(I :\vert :\textrm{Test})$, the following identity holds:

28 $$\begin{aligned} \frac{P(A :\vert :\textrm{Test})\cdot xI}{P(I :\vert :\textrm{Test})\cdot xA } = \frac{A}{I}\,\, . \end{aligned}$$

It is important to interpret the degrees of freedom in this identity. An increase in $P(I :\vert :\textrm{Test})$ leads to a decrease in the total number of tests required to maintain the given *xI*, which may represent a reduction in *xA* or an effort also to increase $P(A :\vert :\textrm{Test})$. Generally, the strategy that focuses on increasing $P(I :\vert :\textrm{Test})$ results in a reduction of $P(A :\vert :\textrm{Test})$, and consequently, *xA*. In other words, if testing symptomatic individuals is prioritized, the testing of asymptomatic infected individuals will decrease.

There is also the strategy of a screening policy that attempts to maximize $P(A+I :\vert :\textrm{Test}) = P(A :\vert :\textrm{Test}) + P(I :\vert :\textrm{Test})$, i.e., it sets a target for how many infected individuals, regardless of whether they have symptoms or not, should be identified per day. With that:

29 $$\begin{aligned} P(A +I :\vert :\textrm{Test}) = \frac{I\cdot xI + A\cdot xA}{N\cdot P(\textrm{Test})} \,\, , \end{aligned}$$

30 $$\begin{aligned} P(\textrm{Test}) = \frac{I\cdot xI + A\cdot xA}{N\cdot P(A +I :\vert :\textrm{Test})} \,\, . \end{aligned}$$

Thus, we define three testing strategies:

- Random testing: $P(I :\vert :\textrm{Test}) = P(I)$, $P(A :\vert :\textrm{Test}) = P(A)$, and $xI = xA = P(\textrm{Test})$.
- Maximization of symptomatic individuals: Testing is restricted to people with symptoms, aiming for targets for $P(I :\vert :\textrm{Test})$ and *xI*.
- Maximization of infected individuals in general: This depends on a good screening policy, aiming for targets for $P(A + I :\vert :\textrm{Test})$, *xA* and *xI*.

In summary, the values of *xI* and *xA*, representing the probability that symptomatic and asymptomatic infected individuals are identified and isolated, can be estimated using Bayes’ theorem. These variables are treated in our model as targets that depend on quantities such as the prevalence of infection in each group, *P*(*I*) and *P*(*A*); the overall probability of being tested $P(\text {Test})$ (determined by policy and availability); and the conditional probability of an individual being infected given a test, $P(I :\vert :\textrm{Test})$ and $P(A :\vert :\textrm{Test})$, also determined by policy. Equations (25) and (26) provide explicit expressions for these estimates, enabling a clear interpretation of testing effectiveness under various strategies.

### Determination of the Basic Reproduction Number $R_0$

The method described in Section 2.1 was applied to the model presented in Section 2.2. The model features 6 compartments of infected individuals, with 16 age groups in each, so that the matrices of interest are of order 96.

The matrix *F*, representing the derivatives of new infection inputs, is given by,31which is broken down into the matrices

32 $$\begin{aligned} (F_E)_{kj} = \frac{\xi _j \beta _{kj}S_{kj}}{N} \,\, , \end{aligned}$$

33 $$\begin{aligned} (F_I)_{kj} = \frac{\beta _{kj}S_{kj}}{N} \,\, , \end{aligned}$$

34 $$\begin{aligned} (F_A)_{kj} = \frac{\alpha _j \beta _{kj}S_{kj}}{N} \,\, . \end{aligned}$$

All other inputs are zero, given that the model only allows new infections arriving in E from E, A or I.

The matrix V, representing the derivatives of transitions between compartments of infected individuals, is given by the matrix below, which is broken down into the matrices given by equations 36 to 47.35

36 $$\begin{aligned} (V_E)_{kj} = \mu _{eq, k} + a_k \,\, , \end{aligned}$$

37 $$\begin{aligned} (V_{A,E})_{kj} = -a_i (1-\rho _i) \,\, , \end{aligned}$$

38 $$\begin{aligned} (V_A)_{kj} = \mu _{eq, k} + \gamma ^{R,A}_{i} + \gamma ^{Qa,A}_{i} \,\, , \end{aligned}$$

39 $$\begin{aligned} (V_I)_{kj} = \mu _{eq, k} + \gamma ^{H}_{k} + \gamma ^{R, I}_{k} + \gamma ^{Qi, I}_{k} \,\, , \end{aligned}$$

40 $$\begin{aligned} (V_{I,E})_{kj} = -a_i\cdot \rho _i \,\, , \end{aligned}$$

41 $$\begin{aligned} (V_{Qi})_{kj} = \mu _{eq, k} + \gamma ^{R,Qi}_{k} + \gamma ^{H,Qi}_{k} \,\, , \end{aligned}$$

42 $$\begin{aligned} (V_{Qi,I})_{kj} = -\gamma ^{Qi}_{k} \,\, , \end{aligned}$$

43 $$\begin{aligned} (V_{Qa,A})_{kj} = -\gamma ^{Qa}_{k} \,\, , \end{aligned}$$

44 $$\begin{aligned} (V_{Qa})_{kj} = \mu _{eq, k} + \gamma ^{R,Qa}_{k} \,\, , \end{aligned}$$

45 $$\begin{aligned} (V_{H, Qi})_{kj} = -\gamma ^{H,Qi}_{i} \,\, , \end{aligned}$$

46 $$\begin{aligned} (V_{H,I})_{kj} = -\gamma ^{H,I}_{i} \,\, , \end{aligned}$$

47 $$\begin{aligned} (V_H)_{kj} = \mu _{eq, k} + \mu _{cov, k} +\gamma ^{H,R}_{i} \,\, . \end{aligned}$$

Finally, with the matrices assembled, $R_0$ is obtained as the spectral radius of $FV^{-1}$. In this work, the C++ high-level library *Eigen* was used to compute numerically the spectral radius.

### Numerical Simulations

Four simulations regarding testing are proposed next. The first simulation aims to verify the effect of varying the parameters *xI* and *xA* on the basic reproduction number $R_0$. The calculation of $R_0$ is executed by varying *xI* and *xA* from 0 to 0.98 in 100 steps for the four contact matrices analyzed in each intervention scenario. The goal is to find the efficient frontier: (*xI*, *xA*) necessary to achieve $R_0=1$. Values of *xI* and *xA* that approach 1 are not used to avoid division by zero in equations 23 and 24, which should not hinder the generality of the conclusions, since having these parameters very close to 1 is not feasible.

Additionally, for each configuration of *xI* and *xA*, the value of critical relative susceptibility was sought: how many times the population would have to be more or less susceptible to achieve $R_0=1$. It can also be interpreted as the scaling factor $\varrho _j$ that must multiply $p_i$ uniformly across all age groups such that $R_0 = 1$ under scenario $j$. For each combination (*xI*, *xA*), the critical value of this factor is determined for which $R_0=1$.

It is noted that the variation of the susceptibility can have different sources. An unlikely acquired immunity in the population would reduce this factor. More likely, however, increased caution in contacts due to general measures adopted during and after the outbreak – such as the use of masks and the habit of handwashing – also leads to a global reduction in susceptibility that can be represented by this relative factor. Thus, it is a factor that is difficult to measure but can be obtained through optimization based on real data of infections and hospitalizations or through extrapolation from controlled experiments. Furthermore, it could represent model error in infections.

Anyway, a high value, above 1, of critical relative susceptibility is a good sign: if the population is more susceptible than initially thought, there would still be a margin of safety. Even just below 1, such as a value around 0.8, indicates that with some effort in daily contacts, it is possible to contain the pandemic. However, a low value, such as 0.5, indicates that it is necessary to reduce the risk of contagion by contact by half for effective protection.

It is then desired to migrate from a purely parametric analysis to understanding the effect of testing on the overall outcome of the epidemic. The capacity to reduce the total number of deaths, after 400 days of the epidemic, will be analyzed for different combinations of *xA* and *xI*, and for each day of the start of testing, regardless of achieving $R_0=1$.

Finally, using equation 27, it is possible to determine the number of tests needed per day of the pandemic to achieve a given (*xI*, *xA*). This is the main point that connects modeling to public policy and allows evaluating the cost to achieve a specific parameter or which (*xI*, *xA*) is feasible given a maximum cost.

Calculations were performed for the three testing strategies described: random testing with $P(\textrm{Test})=xI=xA=0.3$, maximization of symptomatic individuals with $P(I :\vert :\textrm{Test}) = 0.5$ and $xI=xA=0.3$, and maximization of infected individuals with $P(A+I :\vert :\textrm{Test}) = 0.5$ and $xI=xA=0.3$. Each will be applied to the four intervention scenarios, always considering the start on day 10 of the outbreak.

## Results

### Variation of $R_0$ with *xI* and *xA*

**Fig. 2:**
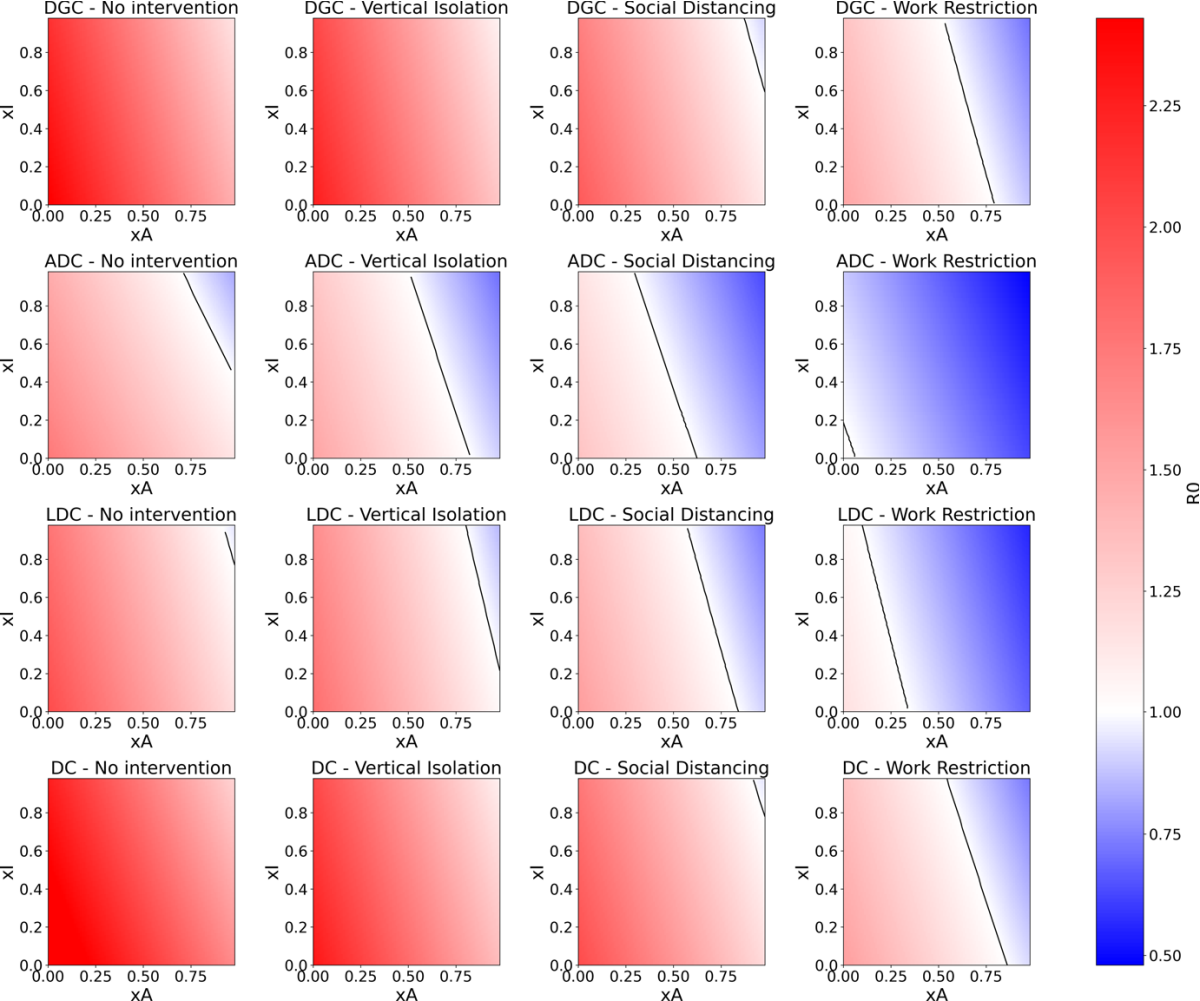
Evolution of $R_0$ with *xI* and *xA* for the contact matrices of DGCs, LDCs, ADCs and DCs, with efficiency frontiers in black

The dependence of $R_0$ with *xI* and *xA* for the four intervention scenarios with the analyzed countries can be seen in Figure 2. The black line indicates the efficient frontier for which $R_0=1$ is observed. The result clearly reflects what is expected from theory: the increase in testing reduces the value of $R_0$, in some cases potentially making it less than 1.

It is observed that increasing the level of isolation allows achieving the same effect with much lower values of *xI* and *xA* (closer to the origin), making it easier to obtain effective mass testing. Furthermore, it is often not possible to reach $R_0=1$ by only increasing testing of symptomatic individuals (moving vertically on the graph). This indicates that identifying asymptomatic individuals is essential for disease control, meaning that testing people with symptoms is not sufficient as a public policy. The difficulty with this measure is identifying asymptomatic individuals since they do not suspect they are infected. Therefore, contact tracing and testing incentive policies are necessary for an effective epidemic containment strategy.

It is also observed that the demographic pattern has a strong impact on the pandemic control strategy. In ADCs and LDCs, pandemic control is possible with a high testing rate, even without interventions. In contrast, for DGCs and DCs, vertical isolation is insufficient, and social distancing is necessary – along with likely restrictions on work – to make the testing rate feasible. Thus, the point already verified in Ridenti et al. (2023); Hilton and Keeling (2020) is reinforced: less developed countries and aged developed countries are the most resistant to the pandemic. This is due to the low average number of contacts in aged developed countries. By isolating the most susceptible individuals, it is possible to stop the epidemic. On the other hand, in less developed countries, the low $R_0$ is due to the shape of the age pyramid, with a majority of young individuals – and therefore less susceptible. In this case, due to the high number of contacts, vertical isolation may not be sufficient to achieve a reasonable testing rate – it is observed that the efficient frontier is located further from the origin than in the case of ADCs.

Despite this similarity between aged developed countries and least developed countries, it is important to keep in mind that the former have a greater ability to invest in testing policies that take advantage of their demographic structure to suppress the disease. In contrast, the latter would have greater difficulty in developing efficient policies due to economic and technological limitations.

### Critical Susceptibility

**Fig. 3:**
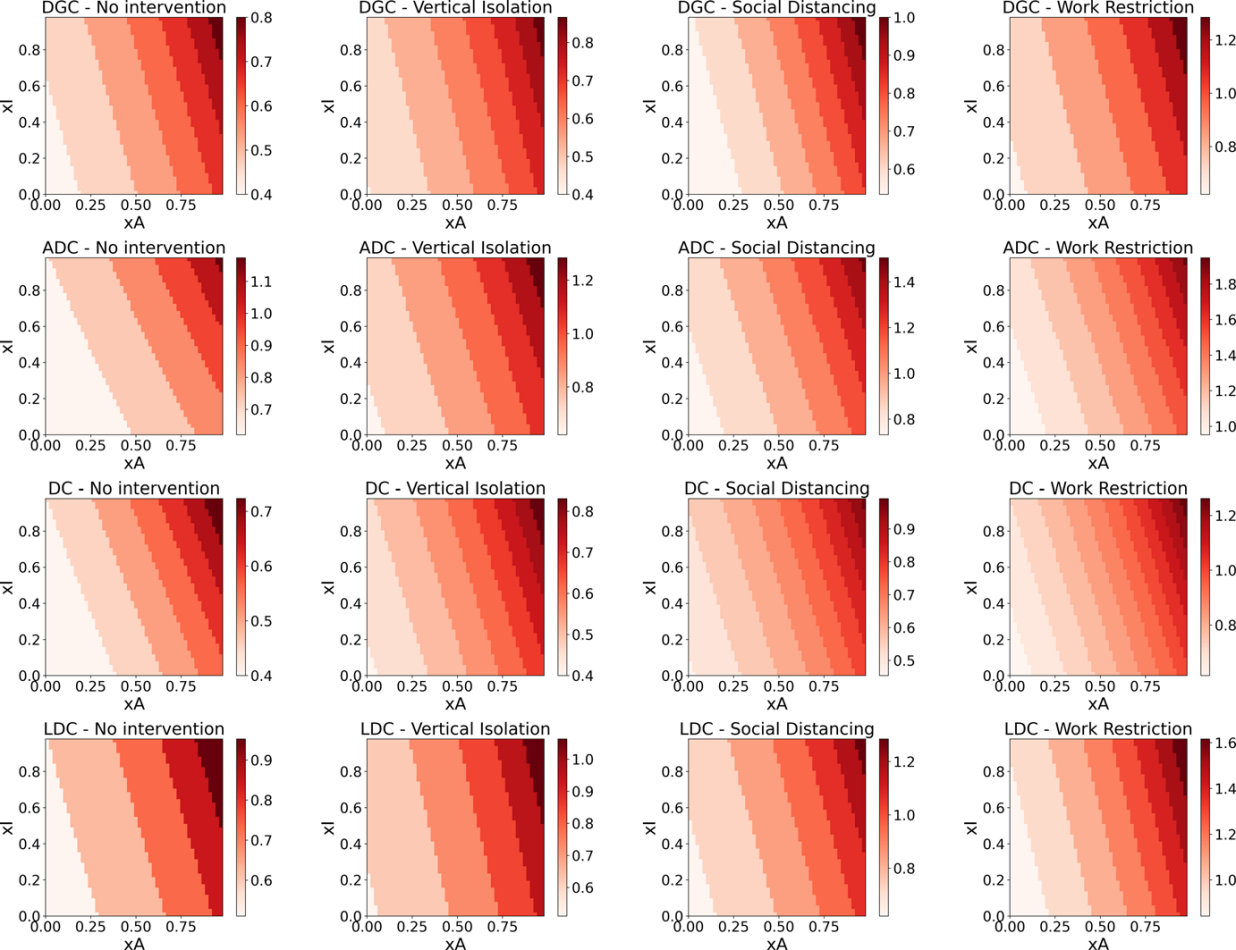
Critical susceptibility as a function of *xI* and *xA* for the contact matrices of DGCs, LDCs, ADCs and DCs

The Figure 3 shows the critical relative susceptibility for each combination of country and intervention scenario. It can be seen that DGCs and DCs start, without interventions and without testing, with a critical relative susceptibility of 0.4. Even with social distancing, the initial values remain around 0.5. This means that, for control without intervention, it would be necessary to reduce the probability of a contact resulting in infection by more than half. On the other hand, the value for ADCs starts around 0.6 and drops to 0.8 with social distancing. An 80% reduction through the use of masks and hygiene practices is more feasible.

Again, LDCs and ADCs present similar numerical situations but a profound difference in reality. While the developed country finds it easier to generate a reduction in susceptibility, the least developing country may lack the resources and infrastructure to do so. For DGCs and DCs, a combination of factors including isolation, measures to reduce susceptibility, and testing proves to be essential.

### Time to Start Mass Testing

**Fig. 4:**
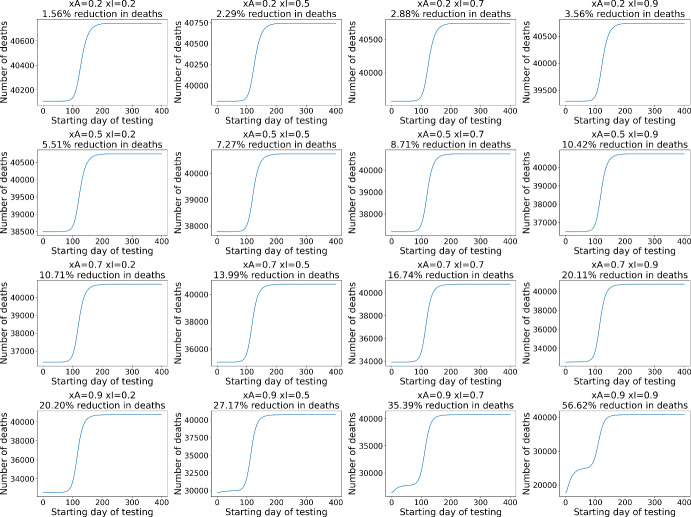
Number of deaths at the end of the disease for each starting day of testing

Figure 4 shows the simulation of the number of deaths for each day of the start of testing. Due to space constraints, the simulations are shown only for DGCs. Additionally, the simulations will be presented for the scenario without intervention. In all cases, starting testing on the last day is equivalent to having no tests, so all the graphs end at the same number.

In this context, we emphasize that the objective of this study is not to predict the number of deaths: the parameters have not been optimized for the scenario of any particular country, and all populations are equal to 10 million. The numbers serve as a verification of the effect of testing in the model, and not as a prediction.

It is observed that, even for DGCs, where it is difficult to achieve $R_0=1$, applying tests from the beginning of the outbreak in a no-intervention scenario can save about 7% of lives with $xI=xA=0.5$. The sigmoid shape of many of the curves indicates that there is an optimal time for testing: before the peak of the outbreak. After the peak of infections, testing becomes less effective, as the peak in deaths has also already passed.

Different configurations of *xI* and *xA* naturally present different results, with a more abrupt reduction observed with an increase in *xA* than in *xI*. Specifically, $xA=0.2$ and $xI=0.9$ only reduce deaths by 3.56%, while $xA=0.9$ and $xI=0.2$ reduce them by 20.20%. Again, this highlights that identifying asymptomatic individuals is essential for effective disease control.

### Testing Cost

**Fig. 5:**
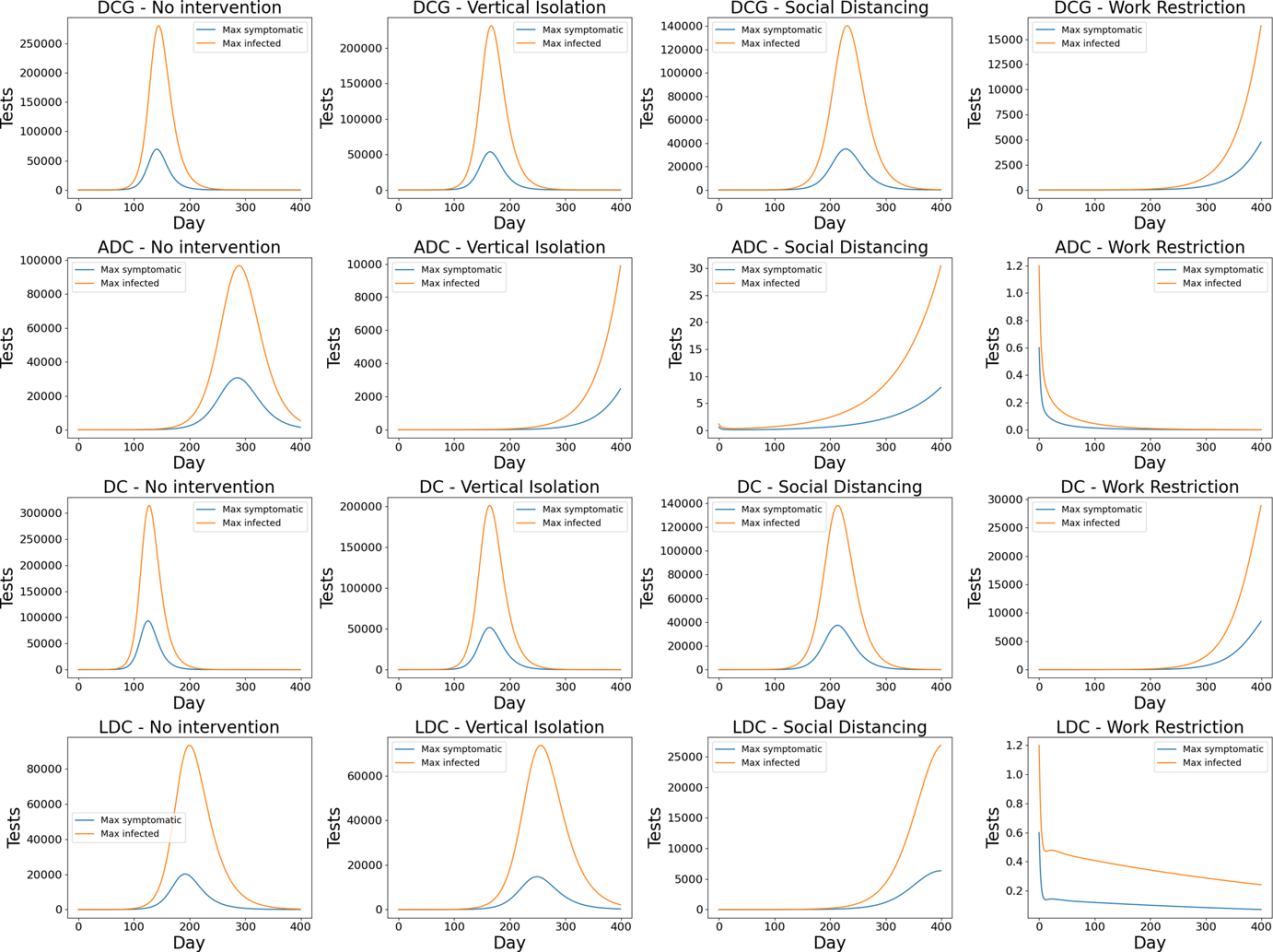
Number of tests needed per day for different isolation scenarios

Figure 5 shows the number of daily tests required to maximize the symptomatic tested and the infected tested. The graph does not include the random testing strategy, as it is a constant function over the days. Table 1 displays the accumulated value over the 400 days shown in the graph for DGCs, i.e., the total number of tests required. With the more intense intervention of work restrictions, the epidemic slows down considerably and does not end by day 400, so the accumulated number of tests does not fully represent the total required and, therefore, was not included in the table.

As the number of tests is directly associated with the number of infected individuals, social isolation provides the same curve-flattening effect that is already known for the number of infections (Ridenti et al. 2023, 2020). It can be seen in Figure 5 that the daily peak of necessary tests for DGCs is reduced from around 250,000 to 140,000 with the strategy of maximizing infected individuals. Additionally, it is noted that for ADCs and LDCs, this case already has $R_0<1$ for the most restrictive social isolation scenario, and the testing policy is not even necessary.

**Table 1:** Total number of tests needed over 400 days of the epidemic for each testing strategy and intevention scenario, in millions of units, for DGCs.

Strategy/Scenario	No intervention	Elderly Isolation	Social Distancing
Random	1200	1200	1200
Focus on symptomatics	3.2	2.9	2.6
Focus on asymptomatics	14.1	13.0	10.5

The strategy of random testing with $xI=xA=P(\textrm{Test})$ is significantly more expensive than the others. Since it does not depend on the number of infected individuals, the number of tests per day is approximately constant at $xI\cdot N$, which, for a normalized population of 10 million, represents 3 million tests per day to achieve $xI=xA=0.3$. Thus, over 400 days, 1.2 billion tests would be used. In practice, this strategy is feasible for countries or cities with a very small population, where it can achieve high levels of *xA* and *xI* without the costs skyrocketing.

In general, the strategies of maximizing symptomatic individuals or maximizing infected individuals are more interesting. Maximizing symptomatic individuals consumes 77% fewer tests over the entire period than maximizing infected individuals, for the scenario without intervention, as shown in Table 1. However, it is well known that public policy rarely manages to maintain a good *xA* in this scenario. Recalling the following relation

48 $$\begin{aligned} xA = \frac{I}{A} \cdot \frac{xI\cdot P(A :\vert :\textrm{Test})}{P(I :\vert :\textrm{Test})}, \end{aligned}$$

the strategy leads to a reduction in $P(A :\vert :\textrm{Test})$. Thus, we fall into the scenario where *xI* increases without varying much in *xA*, which has already been shown to not lead to the best results.

The strategy of setting a target for infected individuals provides better parameters than the previous one. Still, it depends on a good tracking capacity, which has difficult-to-measure costs and is not always feasible, in addition to requiring many tests. Thus, there is a trade-off between the number of tests and the efficiency of epidemic control: the random strategy provides greater direct control over *xI* and *xA* but requires many tests; the infected strategy serves as an intermediate solution, albeit with other hidden costs; and the strategy of maximizing symptomatic individuals being the most feasible of the three.

Another challenge that arises is the monitoring of the ratios $\frac{I}{N}$ and $\frac{A}{N}$, that is, the fractions of symptomatic and asymptomatic infected individuals in the population. While in the simulation we have the model that provides the exact value of each group, decision-makers do not know the actual numbers within the population. To estimate them, epidemiological modeling tools are once again useful, in this case not speculative, but rather aimed at using the correct parameters for the country to obtain valid estimates within a confidence interval. With this, it is possible to estimate, day by day, the number of tests needed.

Finally, it is very important to notice the reduction in the number of tests needed to maintain these standards of *xI* when varying the analyzed intervention scenario. Social distancing reduces the need for tests by 22% for the strategy focused on symptomatic individuals and by 26% for the strategy focused on infected individuals. This is to achieve the same *xI* and *xA*, but previous results show that the need for these parameters itself is reduced as the intensity of the intervention increases. Thus, isolation policies have great potential to lower the need for testing.

Two relevant aspects of COVID-19 were not addressed in our study: reinfection and immunization through vaccination. Vaccination is expected to become a factor only after a significant period – at least a year – so it is reasonable to disregard its impact during the early stages of the epidemic. At the onset of a threatening disease, non-pharmaceutical interventions, such as testing and isolation, are viable options for controlling the spread until vaccines are developed. Reinfection, on the other hand, could play a significant role depending on the virus’s nature, particularly the efficiency and duration of natural immunity. In this study, we assume that reinfection is negligible within the 400-day time frame following the epidemic’s onset. However, we emphasize that this assumption does not always hold true. Additional simulations incorporating reinfection would be necessary to fully assess the effectiveness of isolation measures and testing.

### Caveats and Limitations

This study is subject to several important limitations. First, regarding the *elderly isolation* scenario (Section 2.4.2), we acknowledge that the policy implementation of strict age-based isolation measures may raise ethical concerns and practical limitations. While our model assumes reduced contact rates for individuals over 60 years old, it does not idealize perfect isolation. Instead, a realistic attenuation factor is used to represent partial compliance, especially in settings such as Brazil where multigenerational households are common. As such, the purpose of this scenario is to explore epidemiological consequences of age-targeted interventions rather than advocate for them.

Second, the assumption of 100% test sensitivity (Section 2.6) simplifies the mathematical treatment but does not reflect clinical realities. In practice, test sensitivity—particularly for asymptomatic individuals—is substantially lower and subject to variability based on timing and viral load. Although the model’s testing parameters (*xI* and *xA*) can be interpreted as effective testing probabilities, future versions should incorporate explicit test sensitivity and specificity to better capture the operational limitations of real-world testing strategies.

Third, although the model results point to an optimal testing window prior to the epidemic peak, this should not be interpreted as a vague or heuristic insight. Given adequate epidemiological surveillance—especially estimates of the number of infections over time—the model does allow for quantitative identification of effective intervention windows. Thus, our framework supports data-informed decision-making on the timing of testing implementation, conditional on the availability of timely and accurate infection data.

Finally, the model does not explicitly account for the costs associated with false positives and false negatives, such as economic burdens from unnecessary isolation or undetected transmission chains. These effects could influence both the epidemiological and economic evaluation of different testing strategies. Incorporating such factors, along with behavioral responses to testing outcomes, would provide a more comprehensive basis for public health decision-making.

## Conclusions

Using the SEAHIRQ model, it was possible to develop techniques to compare and evaluate strategies to combat the COVID-19 pandemic, providing a decision-making support model. For the model considered, an analytical method was developed to obtain the reproduction number $R_0$. Additionally, it was found that, with the restrictions considered, having $R_0<1$ is a necessary and sufficient condition to prevent an outbreak of the disease.

This study confirmed the importance of the combination of mass testing and quarantine of the infected with social distancing to contain the spread of the virus. For DGCs and DCs, vertical isolation is not sufficient; horizontal isolation with work restrictions is necessary, combined with testing and measures to reduce susceptibility. ADCs and LDCs theoretically can better execute epidemic control with fewer tests and reduced susceptibility. However, the resources required to implement widespread testing may pose a greater economic burden on the least developed countries compared to the age developed countries, potentially offsetting the advantages offered by their age pyramid.

It was also found that the identification of asymptomatic individuals is essential, and policies such as contact tracing provide greater reduction in the reproduction number and the number of deaths. Different testing distribution strategies present a trade-off between the quality of disease containment and the quantity of tests required. Randomly testing the entire population allows for greater control at a higher cost, while focusing on symptomatic individuals is the cheapest strategy. The use of a tracing policy presents a balance between the number of tests and outcomes for epidemic control, but it has other implementation costs that are difficult to measure and may make it unfeasible. For some strategies, monitoring the state of the epidemic is also necessary, so epidemiological modeling is an indispensable tool for estimating the current situation in terms of public health.

The method used has among its imperfections the assumption that the identified infected individual is isolated perfectly and automatically. However, it is known that this is often not the case – whether due to disbelief, irresponsibility, or economic impossibility. Therefore, it is believed that the population’s commitment to combating the pandemic is an important and difficult-to-measure factor, composed of other subfactors and dependent on the country being evaluated.

In the future, the study can be enriched in different areas: mathematically, a more in-depth analytical study of the equations used may reveal more about the model and its mathematical properties, such as the existence of bifurcations when considering birth rates. Additionally, developing the model to include vaccination and reinfection allows for the analysis of endemic equilibria, new vaccination cycles, and susceptibility reduction, providing relevant conclusions to guide the post-vaccine world.

## Data Availability

The datasets and source code supporting the findings of this study are available from the corresponding author upon reasonable request.
